# Differential disease phenotypes and progression in relapsing–remitting multiple sclerosis: comparative analyses of single Canadian and Saudi Arabian clinics

**DOI:** 10.1186/s12883-021-02317-2

**Published:** 2021-07-27

**Authors:** M. Alluqmani, W. Roda, M. Qqrmli, G. Blevins, F. Giuliani, C. Power

**Affiliations:** 1 Department of Medicine (Neurology), University of Alberta, 6-11 Heritage Medical Research Centre, Edmonton, AB Canada; 2 Department of Medicine, University of Taibah, Medina, Saudi Arabia; 3 Department of Mathematical and Statistical Sciences, University of Alberta, Edmonton, AB Canada

**Keywords:** Relapsing–remitting multiple sclerosis, Geography, Disease progression, Disease modifying therapy, Statistical analyses

## Abstract

**Objective:**

Relapsing–remitting multiple sclerosis (RR-MS) phenotypes differ widely although the variables contributing to this heterogeneity remain uncertain. To assess geographic and ethnic effects on RR-MS phenotypes, we investigated RR-MS patients in Canada and Saudi Arabia.

**Methods:**

A retrospective analysis of patients followed in two MS Clinics was performed in Medina, Saudi Arabia and Edmonton, Canada. Demographic and clinical data were collected for each patient and analyzed using univariable and multivariable statistics. Univariable and multivariable linear regression were used to distinguish the significant clinical and demographic features and neurological systems associated with the change in expanded disability status scale (EDSS) between clinical assessments.

**Results:**

Patients with treated RR-MS were recruited (n = 51, Saudi; n = 47, Canada) although the disease duration was longer in the Canadian cohort (5.6 ± 2.2 yr.) compared to the Saudi cohort (4.4 ± 1.4 yr.) (*P* < 0.05), annual relapse rate and EDSS change were higher in the Saudi cohort (*P* < 0.05). Infratentorial lesion-associated presentation differed (Canada, n = 23; Saudi, n = 13) among groups (*P* < 0.05). Spinal cord lesions on MRI were more frequently detected in Canadian (n = 23) compared to Saudi (n = 1) patients (*P* < 0.05). Patients within the Saudi cohort displayed a significantly greater change in Expanded Disability Status Scale (EDSS) between first and second assessments.

**Conclusions:**

Despite differences in geographic location, ethnicity, and predominance of infratentorial lesions in the Canadian group, the RR-MS phenotypes were similar although the Saudi cohort displayed a more severe disease course.

**Supplementary Information:**

The online version contains supplementary material available at 10.1186/s12883-021-02317-2.

## Introduction

Multiple sclerosis (MS) affects 2.5 million persons globally with increasing recognition of its predominance in women compared to men (3:1) [1]. The phenotypes of MS range widely from a single demyelinating event termed Clinically Isolated Syndrome to a Secondary Progressive phenotype, and in the Western hemisphere relapsing–remitting MS (RR-MS) is the most common phenotype (~ 85%) [2, 3]. Both host genetic and environmental factors contribute to MS disease onset and progression [4]. Although multiple genes have been implicated in the pathogenesis of MS, the MHC complex on chromosome 6 appears to be a key susceptibility domain [5]. Environmental variables including viral infections, vitamin D levels, geographic location, obesity and smoking are also epidemiological factors associated with MS [6].

There is increasing recognition of MS in countries assumed to have low rates of MS, particularly in the Middle East. MS prevalence in Canada is 265–291/100,000 [7]. In comparison, MS prevalence in Saudi Arabia is low at 25/100,000 and RR-MS is the predominant phenotype, primary progressive MS has also be reported [8, 9]. Data from migration studies show that if exposure to a higher risk environment occurs during adolescence (before 15 years of age) the migrant assumes the higher risk of the environment [10]. "Epidemics" of MS have also been reported and these provide further evidence of the importance of environmental factors contributing to MS as reported in the Faroe Islands after WWII [11]. It is uncertain how MS disease phenotypes differ from countries with high prevalence such as Canada or northern Europe compared to Middle Eastern countries with low prevalence although studies of North African patients with MS portend a more aggressive disease course in these patients [12].

We investigated the comparative clinical and radiological aspects of RR-MS in Canada and Saudi Arabia. The working hypothesis was that the severity of the RR-MS disease course and related disease features differed depending on geographic location and ethnicity.

## Methods and Materials

### Clinical design and locations

A cross-sectional prospective analysis of patients receiving active care in university hospital-based MS Clinics was performed in Medina, Saudi Arabia and Edmonton, Alberta Canada. Both MS clinics provide specialized care in terms of diagnosis and treatment of people who are living with MS. The Northern Alberta MS (NAMS) Clinic is affiliated with the University of Alberta, recognized by the Multiple Sclerosis Society of Canada and follows more than 5000 patients. The MS Clinic in Medina serves 1500–2000 patients annually and is associated with the University of Talibah. The study was approved by the University of Alberta Human Biomedical Ethics Committee (Pro00101164) and King Fahad Hospital Ethics Committee.

### Study design

Patient inclusion criteria included the McDonald criteria (2010) for RR-MS (> 3 yr), clinically stable for four weeks pre-study, ability to give consent, ages 18–55, at least one documented relapse in the last year and education greater than Grade 8. Demographic and clinical data were collected for each patient based on two assessments that were one year apart. Exclusion criteria included MS disease-modifying therapies other than interferon-beta, substance abuse or co-morbidities such as terminal cancer or HIV/AIDS.

The demographic and clinical collected data included age, sex, family history of MS or other autoimmune diseases, other co-morbidities, duration of MS diagnosis, relapse rate, results of brain and spinal cord MRIs, disease modifying therapy use, oligoclonal band presence in CSF, education, ethnicity, EDSS and functional score.

The following signs and symptoms were included in the episode categories: limb motor episode (upper limb weakness, lower limb weakness, hemiparesis and spasticity, paraparesis, and walking difficulty); sensory episode (facial sensory symptoms, numbness or sensory impairment in hand/arm and leg/foot, hemibody sensory changes; brainstem episode (vertigo, slurred speech, diplopia, facial muscle weakness, nystagmus, intranuclear ophthalmoplegia); mental episode (cognitive impairment, psychosis and mania); bowel and bladder episode (urinary or fecal retention and incontinence); visual episode (optic neuritis, diplopia); cerebellum episode (gait or limb ataxia); spinal cord episode (spasticity, sensory level); and other episode (fatigue). The following signs were included in the functional scale categories: motor functional scale (generalized weakness, spasticity, motor signs); sensory functional scale (impairment of vibration or and pinprick sensation); brain stem functional scale (brain stem sign, nystagmus, and vertigo); bowel and bladder functional scale (urgency and sensory impairment); visual functional scale (afferent pupillary defect); cerebellum functional scale (dysmetria, dysdiadochokinesis, gait ataxia). All patients underwent a second assessment at one year after the initial assessment for EDSS, functional scores and relapses.

### Statistics

Continuous variables were reported as the mean and standard deviation. Categorical variables were reported with counts and percentages. The Wilcoxon-Mann–Whitney test was used to compare the mean of the two populations for the continuous variables and Fisher’s exact test was used to compare the proportions of the two populations for the categorical variables. Univariable and multivariable linear regression were used to distinguish the significant clinical and demographic features and neurological systems associated with the change in expanded disability status scale (EDSS) between clinical assessments 1 and 2 (EDSS_2_–EDSS_1_) for the patients from Medina (n = 51) and Edmonton (n = 47). The statistical analyses were completed using Stata (version 13.1) and figures were created using R and the R package ‘RColorBrewer’.

Univariable linear regression was used for the patients from Medina and Edmonton separately and the combined group of patients (n = 98), whereas multivariable linear regression was only used on the combined larger group of patients for improved statistical inference and power. Multiple variables (n = 31) were tested for association with the change in EDSS between first and second assessments. For the multivariable linear regression model, all variables were initially included in the model for a hypothesis-driven approach. The variable with the largest *p-*value (greater than 0.05) was identified and the model with and without this variable was compared using the likelihood ratio test. Assuming that the *p-*value from the likelihood ratio test was greater than 0.1, the variable was removed from the model. The final model included 18 variables with a R^2^ value of 0.443 and an adjusted R^2^ value of 0.343. The residual analysis using the Shapiro–Wilk normality test indicated that there is no evidence against the null hypothesis.

## Results

### Clinical and demographic features

Of the 98 recruited patients with RR-MS (n = 51, Medina; n = 47, Edmonton), 40 patients were Caucasian (Edmonton) and 46 patients were Bedouin Arabic (Medina) (Table 1). The female: male ratio was comparable in Edmonton (35:12) and Medina (32:19), as was the age (32.65 ± 7.96 yr., Medina; 34.06 ± 7.77 yr., Edmonton) and age of onset (29.22 ± 7.93 yr., Medina; 29.43 ± 7.82 yr., Edmonton). Most patients had received glucocorticoid therapy in the past (100%, Medina; 78.72%, Edmonton). Although the disease duration was longer in the Edmonton (5.6 ± 2.2 yr.) compared to the Medina group (4.4 ± 1.4 yr.) (*P* < 0.05), total relapses and rate of relapse (total relapses/duration of disease) and annual change in EDSS between Assessments 1 and 2 were higher in the Medina cohort (*P* < 0.05) (Table 1).

**Table 1:** Clinical and demographic features of the Canadian and Saudi cohorts

Variables	Canada (*n* = 47)	Saudi Arabia (*n* = 51)	*P-*value
Age (yr.)	34.06 (7.77)	32.65 (7.96)	NS
Age of onset (yr.)	29.43 (7.82)	29.22 (7.93)	NS
Sex	Female 35 (74.47%) Male 12 (25.53%)	Female (n = 32) (62.75%) Male (n = 19) (37.25%)	NS
Duration of disease (yr.)	5.66 (2.22)	4.43 (1.42)	0.0004
EDSS_1_	1.94 (1.06)	1.33 (0.48)	0.0001
EDSS_2_	1.96 (1.04)	1.56 (0.58)	0.0276
Change in EDSS (EDSS_2_-EDSS_1_)/yr	0.021 (0.232)	0.22 (0.51)	0.0050
Co-morbidities			
Smoking	23 (48.94%)	7 (13.73%)	0.0002
Bronchial asthma	4 (8.51%)	2 (3.92%)	NS
Hypertension	1 (2.13%)	5 (9.80%)	NS
Diabetes mellitus	0 (0%)	4 (7.84%)	NS
Glucocorticoid therapy	37 (78.72%)	51 (100%)	0.0004
Depression	6 (12.77%)	4 (7.84%)	NS
Total relapses	1.51 (1.13)	2.27 (1.11)	0.0002
CSF (OCB)	47 (100%)	51 (100%)	-
Relapse rate/yr	0.26 (0.15)	0.52 (0.21)	< 0.0001
Active inflammation on neuroimaging	1 (2.13%)	0 (0%)	NS

Data are mean (SD) or number (percentage). *EDSS* Expanded disability status scale, *CSF* Cerebrospinal fluid, *OCB* Oligoclonal band. Wilcoxon-Mann–Whitney test was used to compare the mean of the two populations for the continuous variables. Fisher’s exact test was used to compare the proportions of the two populations for the categorical variables

### Neurological disabilities

To determine the type and severity of neurological impairment, the cohorts were compared showing that the risk of brainstem signs and optic neuritis was similar in the Edmonton and Medina cohorts. There were differences between motor, sensory, and cerebellum on the functional scale between Edmonton (motor, 44.68%; sensory, 0%; cerebellum, 19.15%) and Medina (motor, 23.53%; sensory, 21.57%; cerebellum, 0%) (*P* < 0.05), but there were no significant differences between motor, sensory, or cerebellum episodes between Edmonton and Medina. There were differences between bowel and bladder, cerebrum, myelopathy, and fatigue episodes between Edmonton (bowel and bladder, 0%; cerebrum, 17.02%; myelopathy, 48.94%; fatigue, 0%) and Medina (bowel and bladder, 13.73%; cerebrum, 0%; myelopathy, 0%; fatigue, 13.73%) (*P* < 0.05), but there were no significant differences between bowel and bladder on the functional scale between Edmonton and Medina. Neurological systems involved in the disease are also described (Supplementary Table 1).

Based on standard neuroradiological reports obtained for each patient, the MRI findings in each cohort revealed that the spinal cord findings differed between Edmonton (48.94%) and Medina (1.96%) in terms of the presence or absence of lesions (*P* < 0.05). Neuroimaging did not disclose differences in other anatomic sites in terms of the number or type of lesions.

### Change in EDSS between Assessments 1 and 2

**Fig. 1:**
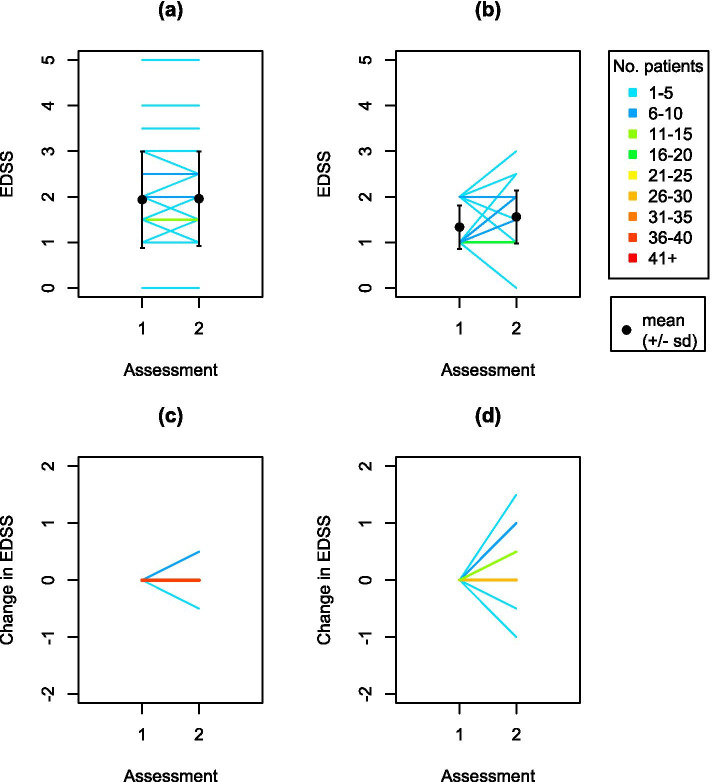
EDSS for patients in Edmonton (**a**) and patients in Medina (**b**). Change in EDSS (EDSS_2_– EDSS_1_) for patients in Edmonton (**c**) and patients in Medina (**d**)

The EDSS at Assessments 1 and 2 are shown for patients from Edmonton (Fig. 1 a) and for patients from Medina (Fig. 1 b). The change in EDSS (EDSS_2_–EDSS_1_) showed that most patients in Edmonton had the same EDSS at Assessments 1 and 2 (Fig. 1 c), whereas the patients from Medina displayed a greater change in EDSS between assessments (Fig. 1 d).

From the univariable linear regression analyses of change in EDSS for the Medina cohort (n = 51), pyramidal episode (coefficient = 0.354, *P* = 0.016), pyramidal functional scale (coefficient = 0.359, *P* = 0.033), and brainstem functional scale (coefficient = 0.382, *P* = 0.007) were all significant and positively associated with change in EDSS. In the univariate linear regression for change in EDSS for the Edmonton patients (n = 47), only cerebellum episode (coefficient = 0.489, *P* = 0.036) was significant and it was positively associated with change in EDSS.

The univariable and multivariable linear regression for change in EDSS for the combined patients from Edmonton and Medina (n = 98) is presented in Table 2. From the univariable analysis, the indicator for geographic location (Edmonton = 0, Medina = 1) was a significant predictor of change in EDSS where those patients from Medina are positively associated with a higher change in EDSS (coefficient = 0.204, *P* = 0.014). A pyramidal episode (coefficient = 0.241, *P* = 0.006) and a brainstem sign on the functional scale (coefficient = 0.200, *P* = 0.018) were significant predictors of change in EDSS and both of these variables were associated with an increase for the change in EDSS. From the multivariable analysis, the Medina cohort (coefficient = 0.410, *P* = 0.001) was significantly associated with a greater change in EDSS after adjusting for age of onset, sex, family history, disease duration, smoking, co-morbidities, lesions on MRI, glucocorticoid therapy, neurological systems involved in episodes and functional scale.

**Table 2:** Univariable and multivariable linear regression for change in EDSS (EDSS_2_–EDSS_1_) with combined patients from Canada and Saudi Arabia (n = 98)

Variables	Univariable			Multivariable		
	**Coef**	**95% CI**	*P-***value**	**Coef**	**95% CI**	*P-***value**
Indicator (Canada = 0, Saudi = 1)	0.204	0.042,0.366	0.014	0.410	0.173, 0.648	0.001
Age of onset (yr.)	0.00311	-	NS	0.0146	0.0043, 0.0248	0.006
Sex (Female = 0, Male = 1)	0.167	-	NS	0.0905	-	NS
Family History (N = 0, Y = 1)	-0.136	-	NS	-0.273	-	NS
Duration of disease (yr.)	-0.0130	-	NS	0.0124	-	NS
Smoking (N = 0, Y = 1)	0.0563	-	NS	0.220	0.029, 0.411	0.025
Asthma (N = 0, Y = 1)	0.130	-	NS	0.269	-	NS
Hypertension/diabetes mellitus (N = 0, Y = 1)	0.0431	-	NS	-0.456	-0.755, -0.156	0.003
Glucocorticoid therapy (N = 0, Y = 1)	0.0307	-	NS	-0.329	-0.645, -0.013	0.041
Motor episode (N = 0, Y = 1)	0.241	0.072, 0.412	0.006	0.567	0.346, 0.787	< 0.001
Sensory episode (N = 0, Y = 1)	0.0224	-	NS	0.298	0.111, 0.485	0.002
Brainstem episode (N = 0, Y = 1)	0.0206	-	NS	0.246	0.026, 0.466	0.029
Mental episode (N = 0, Y = 1)	0.376	-	NS	0.852	0.086, 1.618	0.030
Bowel and bladder episode (N = 0, Y = 1)	0.170	-	NS	0.318	0.013, 0.624	0.041
Visual episode (N = 0, Y = 1)	-0.0160	-	NS	0.161	-	NS
Myelopathic episode (N = 0, Y = 1)	-0.138	-	NS	0.464	0.166, 0.763	0.003
Brainstem functional scale (N = 0, Y = 1)	0.200	0.036, 0.364	0.018	0.356	0.174, 0.538	< 0.001
Visual functional scale (N = 0, Y = 1)	-0.0166	-	NS	0.231	0.022, 0.442	0.031

In the multivariable analysis, age of onset (coefficient = 0.0146, *P* = 0.006), smoking (coefficient = 0.220, *P* = 0.025), motor episode (coefficient = 0.567, *P* < 0.001), sensory episode (coefficient = 0.298, *P* = 0.002), brainstem episode (coefficient = 0.246, *P* = 0.029), mental episode (coefficient = 0.852, *P* = 0.03), bowel and bladder episode (coefficient = 0.318, *P* = 0.041), myelopathic episode (coefficient = 0.464, *P* = 0.003), brainstem functional scale (coefficient = 0.356, *P* < 0.001), visual functional scale (coefficient = 0.231, *P* = 0.031) were all respectively significant and positively associated with change in EDSS, after adjusting for the other variables. Of note, hypertension/diabetes mellitus (coefficient = -0.456, *P* = 0.003) and glucocorticoid therapy (coefficient = -0.329, *P* = 0.041) were significant and negatively associated with change in EDSS after adjusting for the other variables.

## Discussion

The present study compares RR-MS disease variation and progression among patients with full access to health care on two separate continents. Despite geographic and ethno-racial differences between patients in Canada and Saudi Arabia, the RR-MS phenotype was similar with subtle differences in the extent of infratentorial disease. While there was a short period of analysis, there was a significant difference in the disease trajectory with more severe progression in the Saudi group. These findings highlight the potential for delineating factors that contribute to RR-MS progression by comparing different populations.

Implementing the McDonald criteria across different geographic and ethnic boundaries yielded remarkable similarities in disease phenotypes although there are relatively few studies of MS from Middle Eastern centers. Previous reports from the Middle East and Africa including Saudi Arabia describe motor symptoms as the most common presenting symptom followed by sensory changes, optic neuritis and ataxia [8, 13–15]. However, the most frequently presenting symptoms in Lebanon and Libya involved brainstem-cerebellar, followed by sensory, motor, and visual systems [16, 17].

From our data, optic neuritis (55.0% in Canada; 54.9% in Saudi Arabia) and brain stem disease (42.55% in Canada & 52.94% in Saudi Arabia) were common features, either as index or relapse events in both MS cohorts. Sensory symptoms were more frequent among the Saudi group (36.2% in Canada versus 43.1% in Saudi Arabia) and spinal cord, cerebellum and cerebrum disease were more frequent in the Canadian group (48.9%, 19.2%, 17% in Canada), respectively. These differences could be impacted by genetics and western life style factors [18]. Additionally, spinal MRI imaging was more frequently used for the diagnosis in Canada. This might have resulted in relatively higher number of spinal presentations at onset in the Canadian cohort.

In the present data, the age of MS onset was similar for both groups (29.4 years in Canada and 29.2 years in Saudi Arabia) which was consistent with previous reports [16]. However, some studies reported that the mean age at onset in Saudi patients (25.9 years) was lower than that of the non-Saudis (29.4 years) [8]. This finding was also comparable to Western series in which the mean age of onset was 30.6 years in British Columbia [19], 32.9 years in France [20] and 31.4 years from the North American research committee on Multiple Sclerosis registry [21].

The current findings also indicate the female to male ratio for MS was higher in Canada (2.9:1 in Canada versus 1.68:1 in Saudi Arabia). The female to male ratio varies widely in the Arab world: 0.8:1 in Oman, 1.4:1 in Saudi Arabia [8, 14], 1.84:1 in Kuwait [15], 1.33:1 in Qatar [22], 2.85:1 in UAE [23] and 1.2:1 in Iraq [24]. For Saudi and non-Saudi patients the female to male ratio was 1.32:1 and 1.4:1 respectively [8, 25]. In Western series, the female to male ratio is 2:1 or higher [26]. Reports have shown a higher ratio of 3.2:1 in Canadian MS patients [27]. The explanation for the comparatively lower ratios among many Arabic countries are unclear although access to health care and the disease stigma might contribute to the diminished ratio.

In Arab populations, high family aggregation of MS has been reported in Lebanon (5%), Jordan (9.4%), and Qatar (10.4%) [8]. Furthermore, the number of MS patients with positive family histories was 10.6% in Dubai [23] and 2.3% in Egypt [14]. In contrast, Western populations’ family history of MS has been reported to be 15% or higher [28]. In a Danish cohort, the familial lifetime risk for first-degree relatives was 2.5% [29]. In Canada, the familial rate of MS was 17.3% in Saskatchewan [30]. In the current data, there were only two cases with family history in the Canadian and none in the Saudi cohorts. The likely reasons for this low rate of MS family history in MS include the present study design, degree of case ascertainment, and avoidance of sharing history of chronic or debilitating diseases in some cultures.

With respect to disability measures, the mean EDSS score in regional figures were 2.2 in Qatar [22] and 2.43 in Dubai [23]. In our study, the rate of relapses (0.26 in Canada versus 0.52 in Saudi Arabia) and the disability index scores (0.021 in Canada & 0.22 in Saudi Arabia) were higher in the Saudi group. The factors contributing to these differences in disease severity are multifold and they might involve lower adherence to treatment and follow-up, diet (vitamin D, dairy products), early initiation of disease modifying therapies, hypertension, diabetes and comorbidities (viral infections). The mean duration of disease was longer in the Canadian cohort (5.66 years in Canada versus 4.43 years in Saudi Arabia). It is plausible that the Canadian and Saudi groups may have been analyzed at similar stages of the disease course in the present analyses, resulting in the observed differences in disease progression rates.

Most patients in the present Saudi cohort had high educational levels (at least high school). Previous studies have described higher levels of education among MS patients [31]. Implicit in this finding is that the higher the education level, the more likely the patient will seek medical attention and is able to follow up with medical care. In both the Canadian and Saudi cohorts, there were frequent positive oligoclonal bands in CSF (100%). Nonetheless, the likelihood of positive oligoclonal bands in CSF varies widely with 44.2% in Jordanian MS patients [32] and 80.5% in Kuwaiti MS patients [33]. Most Western series report positive oligoclonal band in CSF above 90% in MS patients [34]. A potential reason for the high proportion of MS patients without oligoclonal band in CSF in some cohorts might reflect the former use of gel electrophoresis in the past. Gel electrophoresis has been largely replaced by isoelectric focusing in many countries raising the sensitivity from 50% to over 95% [35]. Thus, these reported differences might be secondary to methodological and diagnostic variations.

Finally, the percentage of patients receiving disease-modifying therapies (DMTs) varies among studies. In an Australian cohort, 65% of RR-MS patients were receiving DMTs, 81.6% had used at least one DMT at some point, and 18.4% had never been treated [36]. In our study, all patients in both cohorts had received a DMT at some point, likely because of government sponsorship of these treatments in both countries. Given the high level of education among MS patients, many patients prefer early initiation of DMT to decrease the number of relapses.

Several limitations in the present study bear mention. The use of different imaging protocols, priorities, and machines among patients might have made radiographic comparisons challenging and potentially open to question in some circumstances. Furthermore, most patients were relatively young and early in the disease course with low EDSS scores, which might constrain the applicability of the findings. In terms of the statistical limitations, the sample sizes for the Saudi and Canada cohorts were 51 and 47 patients, respectively. Given the 31 variables of interest, it was necessary to combine patient data into one larger group of 98 patients for the multivariate analysis and for making robust statistical inferences. Since these patients were receiving active care in MS Clinics in Canada and Saudi Arabia, these results are limited to the specific type of patients that attend these MS Clinics and the small numbers of study subjects, predominantly treated with interferon-beta. Future studies integrating clinical, neuroimaging, and laboratory data from MS clinics around the world including low, middle, and high income countries will enable broader conclusions to be drawn regarding the impact of ethno-cultural and geographic differences on MS phenotypes and their outcomes.

In summary, understanding the differences in phenotypes and disease trajectories for RR-MS in diverse ethnic and geographically discrete populations might provide insights into the factors driving MS onset and progression.

## Supplementary Information

**Additional file 1:** **Supplementary Table 1** Neurological systems involved in Canadian and Saudi groups.

## Data Availability

Data can be made available upon request to the corresponding author.

## References

[1] Harbo HF, Gold R, Tintore M (2013). Sex and gender issues in multiple sclerosis. Ther Adv Neurol Disord.

[2] Marcus JF, Waubant EL (2013). Updates on clinically isolated syndrome and diagnostic criteria for multiple sclerosis. Neurohospitalist.

[3] Vidal-Jordana A, Montalban X (2017). Multiple sclerosis: epidemiologic, clinical, and therapeutic aspects. Neuroimaging Clin N Am.

[4] Sato S, Kira J (2014). Genetic factors in multiple sclerosis and neuromyelitis optica. Nihon Rinsho.

[5] Dyment DA, Ebers GC, Sadovnick AD (2004). Genetics of multiple sclerosis. Lancet Neurol.

[6] O'Gorman C, Lucas R, Taylor B (2012). Environmental risk factors for multiple sclerosis: a review with a focus on molecular mechanisms. Int J Mol Sci.

[7] Rotstein DL, Chen H, Wilton AS, Kwong JC, Marrie RA, Gozdyra P (2018). Temporal trends in multiple sclerosis prevalence and incidence in a large population. Neurology.

[8] Heydarpour P, Khoshkish S, Abtahi S, Moradi-Lakeh M, Sahraian MA (2015). Multiple sclerosis epidemiology in middle east and north africa: a systematic review and meta-analysis. Neuroepidemiology.

[9] Bohlega S, Inshasi J, Al Tahan AR, Madani AB, Qahtani H, Rieckmann P (2013). Multiple sclerosis in the Arabian Gulf countries: a consensus statement. J Neurol.

[10] Gale CR, Martyn CN (1995). Migrant studies in multiple sclerosis. Prog Neurobiol.

[11] Kurtzke JF, Heltberg A (2001). Multiple sclerosis in the Faroe Islands: an epitome. J Clin Epidemiol.

[12] Sidhom Y, Maillart E, Tezenas du Montcel S, Kacem I, Lubetzki C, Gouider R (2017). Fast multiple sclerosis progression in North Africans: Both genetics and environment matter. Neurology.

[13] Tallawy HN, Farghaly WM, Rageh TA, Shehata GA, Badry R, Metwally NA (2013). Door-to-door survey of major neurological disorders (project) in Al Quseir City, Red Sea Governorate. Egypt Neuropsychiatr Dis Treat.

[14] Hamdy SM, Abdel-Naseer M, Shalaby NM, Elmazny AN, Nemr AA, Hassan A (2017). Characteristics and predictors of progression in an Egyptian multiple sclerosis cohort: a multicenter registry study. Neuropsychiatr Dis Treat.

[15] Alroughani R, Ahmed SF, Al-Hashel J (2014). Demographics and clinical characteristics of multiple sclerosis in Kuwait. Eur Neurol.

[16] Sawaya RA, Kanso MI (2009). Multiple sclerosis in Lebanon: a review of 45 cases. Mult Scler.

[17] Yamout B, Barada W, Tohme RA, Mehio-Sibai A, Khalifeh R, El-Hajj T (2008). Clinical characteristics of multiple sclerosis in Lebanon. J Neurol Sci.

[18] Kira J (2003). Multiple sclerosis in the Japanese population. Lancet Neurol.

[19] Tremlett H, Paty D, Devonshire V (2006). Disability progression in multiple sclerosis is slower than previously reported. Neurology.

[20] Confavreux C, Vukusic S, Moreau T, Adeleine P (2000). Relapses and progression of disability in multiple sclerosis. N Engl J Med.

[21] Kister I, Bacon TE, Chamot E, Salter AR, Cutter GR, Kalina JT (2013). Natural history of multiple sclerosis symptoms. Int J MS Care.

[22] Deleu D, Mir D, Al Tabouki A, Mesraoua R, Mesraoua B, Akhtar N (2013). Prevalence, demographics and clinical characteristics of multiple sclerosis in Qatar. Mult Scler.

[23] Inshasi J, Thakre M (2011). Prevalence of multiple sclerosis in Dubai United Arab Emirates. Int J Neurosci.

[24] Al-Araji A, Mohammed AI (2005). Multiple sclerosis in Iraq: does it have the same features encountered in Western countries?. J Neurol Sci.

[25] Daif AK, Al-Rajeh S, Awada A, Al Bunyan M, Ogunniyi A, AbdulJabar M (1998). Pattern of presentation of multiple sclerosis in Saudi Arabia: analysis based on clinical and paraclinical features. Eur Neurol.

[26] Hader WJ, Elliot M, Ebers GC (1988). Epidemiology of multiple sclerosis in London and Middlesex County, Ontario. Canada Neurology.

[27] Orton SM, Herrera BM, Yee IM, Valdar W, Ramagopalan SV, Sadovnick AD (2006). Sex ratio of multiple sclerosis in Canada: a longitudinal study. Lancet Neurol.

[28] Robertson NP, Fraser M, Deans J, Clayton D, Walker N, Compston DA (1996). Age-adjusted recurrence risks for relatives of patients with multiple sclerosis. Brain.

[29] Nielsen NM, Westergaard T, Frisch M, Rostgaard K, Wohlfahrt J, Koch-Henriksen N (2006). Type 1 diabetes and multiple sclerosis: A Danish population-based cohort study. Arch Neurol.

[30] Hader WJ, Yee IM (2014). The prevalence of familial multiple sclerosis in saskatoon, Saskatchewan. Mult Scler Int.

[31] Hammond SR, McLeod JG, Macaskill P, English DR (1996). Multiple sclerosis in Australia: socioeconomic factors. J Neurol Neurosurg Psychiatry.

[32] El-Salem K, Al-Shimmery E, Horany K, Al-Refai A, Al-Hayk K, Khader Y (2006). Multiple sclerosis in Jordan: A clinical and epidemiological study. J Neurol.

[33] Alshubaili AF, Alramzy K, Ayyad YM, Gerish Y (2005). Epidemiology of multiple sclerosis in Kuwait: new trends in incidence and prevalence. Eur Neurol.

[34] Link H, Huang YM (2006). Oligoclonal bands in multiple sclerosis cerebrospinal fluid: an update on methodology and clinical usefulness. J Neuroimmunol.

[35] Mygland A, Trydal T, Vinje BU, Vedeler C (2007). Isoelectric focusing is superior to immunofixation electrophoresis in diagnosing CNS inflammation. Acta Neurol Scand.

[36] Jokubaitis VG, Spelman T, Lechner-Scott J, Barnett M, Shaw C, Vucic S (2013). The Australian Multiple Sclerosis (MS) immunotherapy study: a prospective, multicentre study of drug utilisation using the MSBase platform. PLoS One.

