# Supplementary material for: Differential disease phenotypes and progression in relapsing–remitting multiple sclerosis: comparative analyses of single Canadian and Saudi Arabian clinics
Source: BMC Neurol. 2021 Jul 27;21:295. doi: 10.1186/s12883-021-02317-2 (PMC8314572; doi:10.1186/s12883-021-02317-2)
Supplement: Supplementary file 1 — Additional file 1: Supplementary Table 1 Neurological systems involved in Canadian and Saudi groups. [file 12883_2021_2317_MOESM1_ESM.docx]

Supplementary Table 1

*Differential disease phenotypes and progression in relapsing-remitting multiple sclerosis: comparative analyses of single Canadian and Saudi Arabian clinics.*

M. Alluqmani, W. Roda, M. Qqrmli, G. Blevins, F. Giuliani, C. Power

**Supplementary Table 1:** Neurological systems involved in Canadian and Saudi groups.

| Variables | Canada  (n=47) | Saudi Arabia (n=51) | P-value |
| --- | --- | --- | --- |
| Motor | Episodes 14 (29.79%)  FS 21 (44.68%) | Episodes 19 (37.25%)  FS 12 (23.53%) | NS  0.0334 |
| Sensory | Episodes 17 (36.17%)  FS 0 (0%) | Episodes 22 (43.14%)  FS 11 (21.57%) | NS  0.0006 |
| Brainstem | Episodes 20 (42.55%)  FS 16 (34.04%) | Episodes 27 (52.94%)  FS 25 (49.02%) | NS  NS |
| Bowel and bladder | Episodes 0  (0%)  FS 0 (0%) | Episodes 7 (13.73%)  FS 3 (5.88%) | 0.0129  NS |
| Visual | Episodes 26 (55.32%)  FS 11 (23.40%) | Episodes 28 (54.90%)  FS 15 (29.41%) | NS  NS |
| Cerebellum | Episodes 1 (2.13%)  FS 9 (19.15%) | Episodes 0 (0%)  FS 0 (0%) | NS  0.0009 |
| Cerebrum | Episodes 8 (17.02%) | Episodes 0 (0%) | 0.0020 |
| Spinal Cord | Episodes 23 (48.94%)  MRI 23 (48.94%) | Episodes 0  (0%)  MRI 1 (1.96%) | <0.0001  <0.0001 |
| Other | Episodes 0  (0%) | Episodes 7 (13.73%) | 0.0129 |

Data are number (percentage). FS=functional scale. MRI=magnetic resonance imaging. Fisher’s exact test was used to compare the proportions of the two populations for the categorical variables.
